# Digital Health Experiences of Primary Care Nurses: A Qualitative Meta‐synthesis

**DOI:** 10.1111/inr.70069

**Published:** 2025-07-25

**Authors:** Paloma Robles‐Aguilar, María Dolores Ruiz‐Fernández, Sara Bermudo‐Fuenmayor

**Affiliations:** ^1^ Osuna Health Management Area, Andalusian Health Service, Seville Osuna Spain; ^2^ Department of Nursing, Physiotherapy and Medicine University of Almería Almería Spain; ^3^ Facultad de Ciencias de la Salud, Universidad Autónoma de Chile Providencia Chile; ^4^ Almería Health District, Andalusian Health Service Almería Spain

**Keywords:** digital health, digital health literacy, nursing, primary care, qualitative research

## Abstract

**Aim:**

To analyze primary care nurses’ experiences of integrating and using digital health in their daily practice.

**Background:**

The integration of digital health in primary care, accelerated by the COVID‐19 pandemic, has transformed nursing practices with a view to provide better support and services to patients.

**Introduction:**

The World Health Organization defines “digital health” as the use of electronic technologies to improve health. Its 2020–2025 strategy seeks to integrate these technologies into health systems to facilitate communication between professionals, patients, and authorities. Tools such as telehealth, electronic records, artificial intelligence, and big data are transforming the role of nurses, who must become familiar with them for their performance.

**Methods:**

Qualitative studies on digital health in primary care nursing were reviewed following the Enhancing Transparency in Reporting the Synthesis of Qualitative Research (ENTREQ) guidelines and using the Joanna Briggs Institute for Qualitative Research (JBI‐QARI) criteria.

**Results:**

Eleven articles were analyzed using thematic coding according to Thomas and Harden's approach, identifying three main themes: adaptation to digital health, nurses’ perspective on digital health, and nurse–patient digital interaction.

**Discussion:**

The integration of digital health has required nurses to adapt quickly. They have expressed both benefits and challenges, highlighting the importance of adequate training, personalization in the use of digital tools, information security, and optimization of technological infrastructure.

**Conclusion and implications for nursing and/or health policy:**

It is essential to assess the current competencies of nurses in digital health in order to meet their needs. Health systems should incorporate new technologies into clinical practice guidelines and health programs to improve and update the continuity and quality of care in primary care. Health policies should support the continuing education of nurses and the effective integration of technology.

## 1 Introduction

Digital transformation is a reality in many areas of life, including the healthcare sector (Alarcón Belmonte et al. 2024; Konttila et al. 2019). The incorporation of digital technology in healthcare organizations implies that systems adopt new ways of thinking, executing, and relating to patients and the community, innovating and adapting to a digital culture (Alarcón Belmonte et al. 2024). Thus, the COVID‐19 pandemic has accelerated this transformation in the health sector, with an emphasis on digital health to maintain and promote population health (Benavente‐Rubio 2022).

The World Health Organization (WHO), in its Draft Global Strategy on Digital Health 2020–2025, defines “digital health” as the field of knowledge and practice that focuses on the development and use of electronic technologies to improve people's health (World Health Organization 2021). The main objective of this strategy is the integration of digital technology into health systems, promoting an interoperable digital health environment that facilitates communication between healthcare providers, healthcare workers, patients, public health authorities, and academic and research institutions (Vidal‐Alaball et al. 2023; World Health Organization 2021).

Digital health encompasses a wide range of technologies and applications, including telehealth, electronic health records, artificial intelligence, virtual reality, big data, and robotics (Benavente‐Rubio 2022; World Health Organization 2021). In particular, telehealth is defined as the set of health‐related activities performed remotely with the help of information and communication technologies (ICT) (Ministry of Health and Social Protection, 2020). This modality of remote care between professionals and patients has gained relevance during the COVID‐19 pandemic as a strategy to minimize face‐to‐face consultations and ensure continuity of care (Fernández‐Lasquetty Blanc et al. 2021; Navarro‐Martínez et al. 2024). Within telehealth are telemedicine, tele‐nursing, and teleconsultation, often used interchangeably (Ros‐Navarret 2021).

The introduction of these new ways of working has significantly transformed the roles and responsibilities of nurses, encompassing care practice, teaching, research, and management (Benavente‐Rubio 2022; Fernández‐Lasquetty Blanc et al. 2021). Digital health literacy is the ability to search for, find, understand, and evaluate health information from electronic sources and apply the knowledge gained to address or solve a health problem (Ros‐Navarret 2021). It is essential that nurses are digitally literate, developing the knowledge, skills, and attitudes necessary to effectively use digital technologies in the health sector (Fernández‐Lasquetty Blanc et al. 2021; Soriano 2023). Competencies that nurses need to acquire include skills in information searching and management, ICT‐enabled communication, digital content creation, data protection, and technological problem‐solving (Soriano 2023).

In the primary care setting, nurses must have a thorough knowledge of individuals, families, and the community, as well as the services and resources available in digital health to enhance their professional performance (Marrero 2021). This level of care is a fundamental space for promoting digital health literacy, providing people with educational resources, easy‐to‐use technological tools, and complementary training (Alarcón Belmonte et al. 2024; Lima Serrano et al. 2020). The incorporation of digital health in primary care centers has boosted health education, improved communication between patients and healthcare professionals, and resulted in more efficient monitoring of treatments and patient progress (Vilar Pont et al. 2021). In fact, improvements in health outcomes have been observed in certain chronic pathologies, and the continuity of care between levels of care has been optimized (Ashley et al. 2023; Ekstedt et al. 2023; Navarro‐Martínez et al. 2024; Silva et al. 2022; Vilar Pont et al. 2021). However, there are no studies that analyze the experiences of primary care nurses regarding the integration of digital health in their care work. Therefore, the aim of this qualitative meta‐synthesis is to analyze and describe the experiences of primary care nurses in relation to the integration and use of digital health in daily practice.

## 2 Methods

### 2.1 Design

A qualitative meta‐synthesis was carried out. This methodology involves an interpretative exercise with the aim of increasing the relevance and usefulness of qualitative studies and seeking a more comprehensive interpretation of phenomena (Carrillo González et al. 2008). The purpose was to analyze the experiences of primary care nurses in relation to digital health. The development was guided by the guidelines of the Enhancing Transparency in Reporting the Synthesis of Qualitative Research (ENTREQ) checklist (Tong et al. 2012).

### 2.2 Research Question

A research question was posed following the SPIDER formulation (Cooke et al. 2012). It was “What are the experiences of Primary Care nurses regarding the integration and use of Digital Health in their clinical practices.” The search terms used for the SPIDER search included “Primary care Nursing” for the sample, “Digital health” and “telemedicine” for the phenomenon of interest, “interview” and “focus group” for the design, “experience” for the evaluation, and “qualitative” for the research type.

### 2.3 Search Methods

A search was conducted for qualitative studies published between 2019 and 2024 in English and Spanish. The search period was between February 27, 2024, and April 23, 2024. The databases consulted were PubMed, SCOPUS, CINAHL, and WoS. In addition, the search was completed using gray literature.

### 2.4 Search Strategy

The MeSH terms “Digital Health,” “Experience,” “Primary Care Nursing,” “Telemedicine,” together with the Boolean Operators AND/OR were used to perform the search. Finally, the search strategy used was: (Digital Health OR Telemedicine) AND (Primary Care Nursing) AND Experiences.

### 2.5 Inclusion and Exclusion Criteria

The inclusion criteria for this study were: (a) primary qualitative research articles (descriptive, phenomenological, grounded theory, etc.) or mixed methods; (b) participants had to be primary care nurses; (c) in English or Spanish available in full text; and (d) published from 2019 to 2024. Exclusion criteria were: (a) secondary studies, (b) duplicate articles, (c) articles focused solely on the patient perspective, and (d) focused on nurses without distinction of primary care.

### 2.6 Article Selection Procedure

**FIGURE 1:**
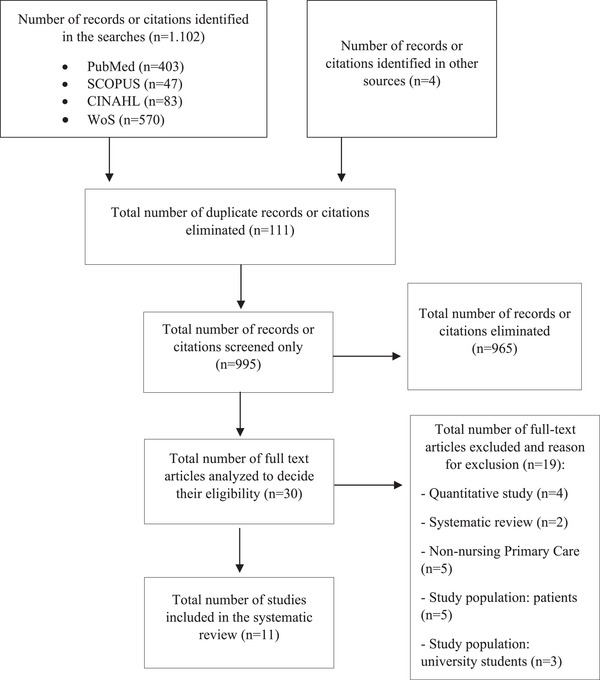
Item selection flowchart.

Article selection was peer‐reviewed by the study investigators in three phases. In the initial phase, a preliminary screening was performed by assessing the titles of the papers retrieved from the databases. Subsequently, in the second phase, duplicate articles were eliminated, and a thorough review of the abstracts of articles that were not discarded in the initial phase was conducted. Finally, in the last phase, a comprehensive reading of the selected articles was carried out to ensure the relevance of their content for the review. During the second and third phases, predefined inclusion and exclusion criteria were applied. In case of discrepancies, a third researcher was consulted to reach a consensus on the suitability of the studies. Each study was assessed manually by the reviewers to ensure methodological rigor. The entire process is illustrated in Figure 1.

### 2.7 Methodological Quality

The Joanna Briggs Institute (JBI) qualitative assessment tool (QARI) was used to assess the methodological quality and risk of bias of the selected studies (Evans et al. 2019). The instrument consists of 10 items, the cutoff point for acceptance of inclusion was 6/10. This tool allows for a rigorous assessment of the quality of the research, thus guaranteeing the reliability, and validity of the results obtained with a qualitative methodological approach. All the articles included in this qualitative synthesis obtained a high score in terms of methodological quality. The results are shown in Supplementary Table S1. The methodological quality control was carried out by two researchers; for this purpose, the concordance between the two evaluators was evaluated, using Cohen's kappa index, the result of which was 0.915.

### 2.8 Confidence in the Review Findings

The Confidence in Evidence from Qualitative Research Reviews (CERQual) criteria were used to assess the level of confidence that could be assigned to the findings (Lewin et al. 2015). This is an innovative and transparent method used in qualitative evidence synthesis that consists of four components: methodological limitations, relevance, consistency, and adequacy of data. We determined confidence in the topics as high, moderate, low, or very low. The results are shown in Supplementary Table S2.

### 2.9 Data Synthesis and Analysis

In this work, the selected studies were subjected to a thematic analysis, which was carried out manually by the three researchers. To synthesize the research, the findings of the included studies were coded line by line, elaborating descriptive themes and generating analytical themes and subthemes. For this purpose, the thematic synthesis method described by Thomas and Harden, which consists of three stages, was followed. The first stage consisted of text coding, in which, based on the question posed, the main findings of the selected studies were reviewed by inductive line‐by‐line coding, resulting in 35 codes representative of the studies analyzed. In the second stage, descriptive themes were developed through the search for similarities and differences between codes, thus grouping them into broader categories, finally identifying 10 subthemes that captured various dimensions of the study. Finally, in the third stage, analytical themes were developed, further analyzing the subthemes by the researchers and concluding in three main themes that provided a deeper understanding of the data (Thomas and Harden 2008).

## 3 Results

### 3.1 Characteristics of Studies

The articles finally selected addressed different areas of digital health in the context of primary care nursing. The main topics included telehealth (*n* = 2), telemonitoring (*n* = 2), digital competencies (*n* = 3), and digital communication (*n* = 4). Countries of origin of the studies include Sweden (*n* = 4), Australia (*n* = 2), Wales (*n* = 1), Norway (*n* = 1), Spain (*n* = 1), Canada (*n* = 1), and Belgium (*n* = 1). Information on the studies is included in Table 1, thus providing a clear and concise synthesis of the relevant information contained in each case.

**TABLE 1:** Characteristics of the included studies.

Author and year	Objective	Desing	Participants	Data collection	Data analysis	Results
Lie et al. 2019	Influence of a digital health intervention based on the Guided Self‐Determination program on the patient–nurse relationship from the nurses’ perspective.	Qualitative descriptive study	4 primary care nurses and 10 patients	Semi‐structured interviews	Graneheim and Lundman	Ambiguous experiences revealed about the Guided Self‐Determination program as it not only facilitates reciprocal understanding and flexibility in the relationship but can also create a more fragile relationship.
Entezarjou et al. 2020	Staff experiences of working with a digital communication platform.	Qualitative descriptive study	10 primary care nurses and 9 primary care physicians	Interviews	Graneheim and Lundman	They suggested 5 categories summarized in 2 themes: “Adjusting to a new media” and “Digitally filtered primary care.”
James et al. 2021	Australian primary care nurses’ experiences of using telehealth during COVID‐19.	Qualitative descriptive study	25 primary care nurses	Interviews	Braun and Clarke	They identified 4 themes: preparedness, telehealth accessibility, experience, and impact on nurses. Telehealth improved access to care.
Carlsson et al. 2022	Nurses’ experiences with person‐centered care and competence after participating in a Digital Competence in Care course.	Qualitative descriptive study	8 primary care nurses	Interviews	Graneheim y Lundman	Four categories: openness to change, striving for requirements management, developing new ways of working, and focusing on remote patients.
Nilsen et al. 2022	Lessons from the COVID‐19 pandemic from nurses and assistants in primary care that can influence the future of primary care.	Qualitative descriptive study	11 registered primary care nurses and 10 nursing assistants	Semi‐structured interviews	Krippendorff	They identified 2 categories: lessons on primary care staff and patient behavior, and lessons on primary care routines (meetings and digital consultations…).
Ashley et al. 2023	Perceptions on the sustainability of telehealth in post‐pandemic primary care.	Qualitative descriptive study	14 primary care nurses, 13 primary care physicians, and 6 hospital care physicians	Semi‐structured interviews	Braun and Clarke	Participants’ insights focused on lessons learned about telehealth and its sustainability, covering financing, security, hybrid services, and access to support.
Ekstedt et al. 2023	Patients’ and health professionals’ experiences and sense of security when using telemonitoring of chronic diseases at home.	Qualitative study with an inductive approach	7 primary care nurses, 2 primary care physicians, and 20 patients	Semi‐structured interviews	Inductive content analysis	Safety in telemonitoring depended on collaboration between patients and healthcare professionals, increasing awareness of symptoms and early detection.
Van Grootven et al. 2023	Experiences of patients and healthcare professionals in the implementation of telemonitoring of patients with COVID‐19.	Qualitative study with pragmatic orientation	13 primary care nurses, 16 physicians, and 17 patients	Semi‐structured interviews and focus groups	QUAGOL	Patients assessed telemonitoring as reassuring, as did healthcare professionals. They faced logistical challenges, financial challenges, lack of digital skills.
Havard et al. 2024	Nurses’ perceptions of digital nursing.	Mixed‐methods approach, quantitative and descriptive qualitative	249 primary care nurses.	25 semi‐structured interviews and a survey of open‐ended and closed questions	Thematic analysis	They identified 4 themes: access, impact on care, technology, and digital future. They highlight positive outcomes in work and patient outcomes.
Navarro‐Martínez et al. 2024	Nursing professionals’ views on the current limitations and future potential of digital health tools in tele‐nursing.	Qualitative descriptive study	68 primary care nurses	Opinions in the online course forum	Braun and Clarke	They value tele‐nursing positively for its benefits and ability to humanize care but highlight limitations such as lack of training and digital health literacy.
Rouleau et al. 2024	Meaning of compassionate virtual care for nurses and how nurses realized compassionate care through virtual interactions in primary care.	Qualitative descriptive interpretative study	18 Primary care nurse practitioners and 2 primary care registered nurses	Semi‐structured interviews	Braun and Clarke	They described compassionate care as fundamental to nursing practice, evolving in virtual interaction and requiring a balance between patient expectations and virtual compassionate care.

### 3.2 Themes and Subthemes

Three main themes, 10 subthemes, and 35 codes were identified, as detailed in Table 2.

**TABLE 2:** Themes, subthemes, and codes.

Themes	Subthemes	Codes
1. Adaptation to digital health in primary care.	1.1 Need for rapid adaptation and change. 1.2 Patient safety. 1.3 Logistical challenges.	Rapid adaptation to digital health, adaptation to telehealth, use of technology as a catalyst, increasing familiarity with digital health, pandemic as a driving force for change, telehealth readiness and accessibility, changes in PC work routines, disrupted PC workflow, maintaining patient safety, hacking of health information, health record vulnerability, lack of technological resources, outdated resources, access to technological support.
2. Digital health from the perspective of primary care nurse.	2.1 Handling of technology and new working methods. 2.2 Need for training in digital health. 2.3 Benefits of digital health in primary care nursing. 2.4 Challenges of digital health in primary care nursing.	Impact on the role of nurses, technological skills, relevance in daily practice, effectiveness of digital meetings, telemonitoring, tele‐nursing, teleconsultation, safety experiences in digital monitoring, fears and benefits of digital communication, new knowledge in digital health, acceptance of the digital society, adjustment to new digital communication media.
3. Digital nurse–patient interaction.	3.1 Evolution of care through virtual interactions. 3.2 Advantages of virtual care. 3.3 Difficulties of virtual care.	Little interaction in telehealth, face‐to‐face versus telehealth, importance of face‐to‐face contact, limited method of communication, advantages of teleconsultations with patients, written communication, reciprocal understanding in the patient‐nurse relationship, feedback in communication, digital divide, focusing on patients at a distance.

#### 3.2.1 Adaptation to digital health in primary care

##### 3.2.1.1 Need for Rapid Adaptation and Change

The COVID‐19 pandemic has acted as a significant driver for digitalization in healthcare, requiring rapid adaptation of nurses, and healthcare personnel in general, to new ways of working (Carlsson et al. 2022). The implementation of digital technologies has been compared with a fast‐moving “digital train” that must be adapted to because of its continuous and accelerated change (Carlsson et al. 2022). For nurses, this rapid digitization was met with fear and uncertainty, but over time, they have adopted a positive view of digital health (Entezarjou et al. 2020). Teleconsultation has undergone exponential development in a short time, so that the use of web platforms for virtual meetings has become normalized, leaving aside physical presence in many settings (Nilsen et al. 2022).

“The pandemic has been the driving force to initiate digitalization. It was a very sudden change that had to be made to maintain attention and contact” (Carlsson et al. 2022).

##### 3.2.1.2 Patient Safety

The incorporation of digital health has raised significant concerns regarding patient safety (Navarro‐Martínez et al. 2024). The fact of using applications and programs for teleconsultations puts the safety and privacy of the patient and the professional at risk (Navarro‐Martínez et al. 2024).

“The main problem I find is that today, in order to carry out telecare, we must resort to various applications or programs from our own electronic devices, which puts the safety and privacy of patients and ourselves at risk” (Navarro‐Martínez et al. 2024).

##### 3.2.1.3 Logistical Challenges

Digitalization in health care presents logistical challenges that are present in the adaptation to digital health. In primary care, there are a wide variety of resources that facilitate telehealthcare for appointments, follow‐ups, counseling, health education, such as computers, corporate e‐mails, webcams, telephones, applications. However, some of these resources are outdated and delay digital tasks. In addition, another perceived challenge is the lack of resources on the part of the patient (Navarro‐Martínez et al. 2024). Other studies refer to inability to log on to the computer as a simple but recurring problem, which can also pose a barrier to service delivery (Carlsson et al. 2022).

“I believe that currently, the biggest barrier to ICT use in practice is material resources. Specifically, those that are outdated and delay many of the tasks we want to carry out digitally” (Navarro‐Martínez et al. 2024).

#### 3.2.2 Digital Health From the Perspective of Primary Care Nurses

##### 3.2.2.1 Technology Management and New Work Methods

Primary care nurses have experienced a significant adaptation to the management of technologies and new working methods, learning that it is not something to be afraid of (Ashley et al. 2023). These new working methods are seen as a way in which all the necessary tools and information are gathered in one place, optimizing nursing care (Havard et al. 2024). Many professionals had never used the necessary tools in digital health before, so they have had to familiarize themselves with them (James et al. 2021).

“I think we've learned that it's something not to be afraid of…especially in PA, I know nurses have been wanting it for a long time” (Ashley et al., 2023).

##### 3.2.2.2 Need for Training in Digital Health

The implementation of digital health has led to the need for training of nurses, which is present in several studies. The importance of taking training courses and reading relevant and truthful information on digital health is highlighted (Carlsson et al. 2022). In another study, although the importance of adequate training is highlighted, there is a lack of knowledge of the resources available for this purpose (Ashley et al. 2023). Allusion is also made to the importance of training and education not only of the professional but also of the patient (Navarro‐Martínez et al. 2024).

“I'm not sure what's available…I could probably use a lot of training. I haven't really looked for it or had time to look for it” (Ashley et al. 2023).

##### 3.2.2.3 Benefits of Digital Health for Primary Care Nurses

The benefits of digital health from the perspective of nursing staff are reflected in multiple areas, as evidenced by the studies included. They refer to the fact that the integration of tele‐nursing not only facilitates communication within the multidisciplinary team but also opens doors to areas that are difficult to access, reducing waiting lists and improving the quality of care (Navarro‐Martínez et al. 2024). The same study highlights the reduction of unnecessary transfers and the decrease in morbidity and mortality (Navarro‐Martínez et al. 2024). Another study points out, as a benefit, the observation of patients in their home environment, which reveals aspects of their daily life that may go unnoticed in a face‐to‐face consultation (Rouleau et al. 2024). Ekstedt et al. (2023) highlight the benefits of home monitoring in patients who are newly diagnosed with chronic diseases and need to learn about their illness.

“Tele‐nursing facilitates the work of the multidisciplinary team and allows information to reach areas that are more difficult to access, shortening waiting lists” (Navarro‐Martínez et al. 2024).

##### 3.2.2.4 Digital Health Challenges for Primary Care Nurses

Primary care nurses face significant challenges in the integration of digital health. Resilience and personal growth are essential in order to cope with technologies, even if this has never been done before (Ashley et al. 2023). Carlsson et al. (2022) refer to the need to deal with new digital systems as a challenge for professionals in their training in digital competences (Carlsson et al. 2022). The importance of creating standards as a guide for the correct use of telehealth is highlighted (Ashley et al. 2023). Another article also refers to the need for the creation of protocols due to the changing needs and lack of training of nurses in digital health (Navarro‐Martínez et al. 2024).

“Digital competence involves daring to deal with new digital systems and challenging myself and my co‐workers” (Carlsson et al. 2022).

#### 3.2.3 Digital Nurse–Patient Interaction

##### 3.2.3.1 Evolution of Care Through Virtual Interactions

Healthcare through virtual interactions has led to changes in the nurse–patient relationship, as referred to in several articles included in this meta‐synthesis. In Carlsson et al. (2022), the use of teleconsultation raises the question “for whom is this an option, and when does it fit in?” Furthermore, they suggest that teleconsultation should be seen as a complement to face‐to‐face consultation, rather than a substitute (Carlsson et al. 2022).

“Last week I met a patient in palliative care, and she was interested in digital meetings and said she would prefer to sit at home with her family. You have to take the time to think: for whom is this an option? And when does it fit? No solution can be a generalization” (Carlsson et al. 2022).

##### 3.2.3.2 Advantages of Virtual Care

In the selected articles, nurses have developed a series of advantages of virtual care that have a positive impact on them as well as on patients and their families. They highlight the ease of access to the consultation for those who are used to digital interactions, such as young people (Navarro‐Martínez et al. 2024). This saves time, as they can connect from anywhere, which facilitates the integration of consultations into everyday life without the need to travel (Nilsen et al. 2022). They also report benefits for patients with chronic diseases and in palliative care, as more accessible care can be maintained and continuous accompaniment can be provided (Rouleau et al. 2024). For nurses, this offers them the possibility of capturing details that would not be evident in a traditional consultation, such as living conditions, or family dynamics, which enriches the diagnosis and understanding of the patient's context. On the other hand, the option to communicate in written form virtually gives patients the ability to elaborate on what they wish to share (Lie et al. 2019).

“I think we should continue with digital consultations. There are many patients who are very happy with it, they can sit at work and maybe have a teleconsultation during lunch instead of having to leave. It also saves time for patients” (Nilsen et al., 2022).

##### 3.2.3.3 Difficulties of Virtual Care

Several important difficulties have also been reported in the nurse–patient relationship. They indicate that a large proportion of patients prefer face‐to‐face consultations (Ashley et al. 2023). They highlight the lack of personalization of digital interactions as a concern (Carlsson et al. 2022). Another barrier developed has been the digital divide, which affects older people, who may have technological difficulties necessary for this virtual care (James et al. 2021). This gap is also present in rural areas (Navarro‐Martínez et al. 2024). With regard to written virtual communication, there are limitations in emotional expression and in the subtlety of verbal language, which hinders the effective transmission of messages and emotional support (Lie et al. 2019). They also highlight the reluctance of health professionals themselves, who opt for face‐to‐face consultation (Navarro‐Martínez et al., 2024).

“In any given week, 10% of my patients may choose telehealth and 90% choose face‐to‐face” (Ashley et al., 2023).

## 4 Discussion

The main objective of this qualitative meta‐synthesis was to analyze and synthesize the experiences of primary care nurses regarding the integration and use of digital health in their daily practice. The incorporation of digital health was initially challenging due to the rapid adaptation required by the COVID‐19 pandemic, and nurses have identified significant benefits in their daily practice. However, they perceive difficulties too.

The incorporation of digital health in the field of primary care experienced a significant boost due to the COVID‐19 pandemic, marking the beginning of an ongoing transformation in ways of working (Carlsson et al. 2022). The results obtained coincide with those presented in the study by Vilar‐Pont et al. (2021), and further evidence is needed on the telematic interventions that can be applied to the available digital media that can become standard practice in primary care (Vilar Pont et al. 2021).

The adoption of digital health is a challenge for nurses, and although it is generally valued as positive in this study, some barriers to its use have been identified, such as dependence on the internet, mobile networks, and technological equipment, as well as the use of outdated technology and the lack of adequate material resources (Navarro‐Martínez et al. 2024). This has also been reflected in a study that analyzed the implementation of telehealth and where it led to an overload on professionals, generating interruptions in the care received by patients (Aijaz et al. 2024). In the study by Nyoni et al. (2023), one of the main perceived barriers was related to logistics, such as lack of access and availability, which resulted in longer response times for patients (Nyoni et al. 2023). Another major drawback reflected in the present research was maintaining the safety and privacy of patients and users, as in other research (Navarro‐Martínez et al. 2024). In this sense, nurses have a great role to play in maintaining and ensuring cybersecurity principles to avoid exposure and misuse of sensitive information (Benavente‐Rubio 2022).

On the other hand, the benefits of digital health from the nursing perspective are evident in multiple areas, such as the reduction of morbidity and mortality, the prevention of hospitalizations, the ease of multidisciplinary teamwork, and access to information in remote and hard‐to‐reach areas (Navarro‐Martínez et al. 2024). These results are consistent with other research, which highlights the reduction of response times, adverse events, and optimization of resources (Jimenez et al. 2020). Thus, one of the aspects has been home monitoring, which allows nurses to carry out continuous and personalized follow‐up of the patient, managing their health more effectively from home and increasing patient empowerment (Ekstedt et al. 2023; Jimenez et al. 2020). Benavente‐Rubio (2022) points out that this system allows care to be transformed, opening a window to achieve “precision nursing” (Benavente‐Rubio 2022).

Despite the benefits, the need for nursing education has been a recurring deficit. Lack of time and lack of knowledge about available training opportunities, together with the need for theoretical and practical training, and the importance of developing comprehensive and accessible educational programs to improve clinical practice and patient care, are some of the obstacles (Ashley et al. 2023; Carlsson et al. 2022; Navarro‐Martínez et al. 2024). Jiménez et al. (2020) categorize digital health literacy as one of the major difficulties among primary care health workers, especially nursing professionals (Benavente‐Rubio 2022). Several studies have even highlighted the importance of assessing the levels of digital health literacy among university nursing students and the need to incorporate it into university curricula, given the potential impact this has on the training of future professionals (Kleib et al. 2024; Stellefson et al. 2011). Key areas of competence in digitization, such as knowledge of technology, digital skills to deliver good patient care, social and communication skills, and ethical considerations, are fundamental (Konttila et al. 2019). In addition, healthcare professionals must be motivated to gain experience in digitization in their professional context in order to address these challenges effectively (Konttila et al. 2019).

In terms of primary care nurse–patient interaction, the results show that a minority of patients opt for telehealth (Ashley et al. 2023), stating that digital consultations may affect the quality of the interaction, so they prefer face‐to‐face contact (Carlsson et al. 2022). Even a percentage of health professionals prefer face‐to‐face consultation due to concerns about the quality of care and the challenges inherent in digital interactions (Silva et al. 2022). The digital divide is a major barrier, especially among older patients and those located in rural areas with difficult access to digital systems (James et al. 2021; Navarro‐Martínez et al. 2024). Vidal‐Alaball et al. (2023) stress the importance of developing ICT research clusters in rural or remote areas to enable the development of technologies that facilitate telehealth and thus reduce the rural digital divide.

However, the advantages of digital interaction between primary care nurses and patients are significant, supporting its value as an effective tool in healthcare. Nurses highlight that it fosters patient autonomy and facilitates compassionate care and continuous contact in the management of chronic and palliative diseases (Navarro‐Martínez et al. 2024; Rouleau et al. 2024). Teleconsultation, in addition to saving time, allows access to care from anywhere (Nilsen et al. 2022). The possibility of maintaining visual contact through the screen is essential for patients who require recurrent consultations, humanizing the profession and providing support (Navarro‐Martínez et al. 2024; Rouleau et al. 2024; Van Grootven et al. 2023). These advantages are in line with those highlighted by Silva et al. (2022), who emphasize the promotion of patient autonomy, time, and cost savings due to no need for travel, as well as the rapid detection of clinical changes at home (Ashley et al. 2023; Silva et al. 2022). Carlsson et al. (2022) raise the question: “to whom is this an option? And when does it fit?,” highlighting the importance of personalizing digital care according to the needs and preferences of each patient (Carlsson et al. 2022).

### 4.1 Limitations

Firstly, most of the studies focused on digital health from the perspective of telehealth, even though digital health encompasses other aspects and resources relevant to the daily practice of primary care nurses. However, this meta‐synthesis has allowed us to obtain a global view on the experiences of primary care nurses in the use of digital tools. Secondly, some of the studies have involved patients and other health professionals alongside nurses. Even so, this variability has allowed this research to be richer. Finally, the methodological approach used in most of the studies has been the descriptive qualitative design; there are no studies using a phenomenological approach or grounded theory. Despite this low variability of approaches, descriptive qualitative studies help to explore and understand the experiences of primary care nurses.

## 5 Conclusion

The integration of digital health into the daily practice of primary care nurses has been an adaptation for both nurses and patients, with benefits such as improved communication and follow‐up. Although telehealth brings advantages such as convenience and continuity of care, the preference for face‐to‐face contact remains common. Nurses highlight the need for continuous and adequate training to optimize these systems, but point out that it is often insufficient, highlighting the importance of developing proper digital health literacy. In addition, the implementation of these resources must be personalized according to the patient's needs, ensuring information security and relying on an adequate technological infrastructure.

### 5.1 Implications for Nursing and Health Policy

Health systems must design training programs in digital competencies for primary care nurses. It is essential to analyze and assess the current competencies of nurses in this field, which will allow more personalized and relevant training to be designed to meet their specific needs. Healthcare systems should consider incorporating new technologies and their effective use in clinical practice guidelines and health programs to improve the continuity and care of patients in primary care centers. Their use will encourage prevention and health promotion in individuals, families, and communities. In addition, health policies should support the continuing education of nurses and the effective integration of technology to strengthen the role of nursing and improve health care.

## Author Contributions

Study conception: P R‐A, M.D. R‐F, and S B‐F. Study design: P R‐A, M.D. R‐F, and S. B‐F. Data collection: P R‐A, S. B‐F. Data analysis: P R‐A and S. B‐F. Drafting of manuscript: P R‐A, M.D. R‐F, and S. B‐F. Study supervision: M.D. R‐F. Critical revisions for important intellectual content: M.D. R‐F. Administrative support: P R‐A, M.D. R‐F, and S. B‐F. Technical support: P R‐A, M.D R‐F, and S. B‐F. Material support: P R‐A, M.D R‐F, and S. B‐F.

## Conflicts of Interest

The authors declare no conflicts of interest.

## Supporting information




**Table S1**. JBI‐QARI methodological assessment
**Table S2**. CERQual assessments.
