# Supplementary material for: Digital Health Experiences of Primary Care Nurses: A Qualitative Meta‐synthesis
Source: Int Nurs Rev. 2025 Jul 25;72(3):e70069. doi: 10.1111/inr.70069 (PMC12291427; doi:10.1111/inr.70069)
Supplement: Supplementary file 1 — Table S1. JBI‐QARI methodological assessment Table S2. CERQual assessments. [file INR-72-0-s001.docx]

**Supplementary material.**

**Table S1.** JBI-QARI methodological assessment.

|  | Ashley et al., 2023 | Carlsson et al., 2022 | Ekstedt et al., 2023 | Entezarjou et al., 2020 | Havard et al., 2024 | James et al., 2021 | Lie et al., 2019 | Navarro-Martínez et al., 2024 | Nilsen et al., 2022 | Rouleau et al. 2024 | Van Grootven et al., 2023 |
| --- | --- | --- | --- | --- | --- | --- | --- | --- | --- | --- | --- |
| Is there congruity between the stated philosophical perspective and the research methodology? | Yes | Yes | Yes | Yes | Yes | Yes | Yes | Yes | Yes | Yes | Yes |
| Is there congruity between the research methodology and the research question or objectives? | Yes | Yes | Yes | Yes | Yes | Yes | Yes | Yes | Yes | Yes | Yes |
| Is there congruity between the research methodology and the methods used to collect data? | No | Yes | Yes | Yes | No | No | Yes | No | Yes | Yes | No |
| Is there congruity between the research methodology and the representation and analysis of data? | Yes | Yes | Yes | Yes | Yes | Yes | Yes | Yes | Yes | Yes | Yes |
| Is there congruity between the research methodology and the interpretation of results? | Yes | Yes | Yes | Yes | Yes | Yes | Yes | Yes | Yes | Yes | Yes |
| Is there a statement locating the researcher culturally or theoretically? | Yes | Yes | Yes | Yes | No | Yes | Yes | Yes | Yes | Yes | Yes |
| Is the influence of the researcher on the research, and vice- versa, addressed? | Yes | Yes | Yes | Yes | Yes | Yes | Yes | Yes | Yes | Yes | Yes |
| Are participants, and their voices, adequately represented? | Yes | No | Yes | No | No | Yes | Yes | No | Yes | No | Yes |
| Is the research ethical according to current criteria or, for recent studies, and is there evidence of ethical approval by an appropriate body? | Yes | Yes | Si | Yes | Yes | Yes | Yes | Yes | Yes | Yes | No |
| Do the conclusions drawn in the research report flow from the analysis, or interpretation, of the data? | Yes | Yes | Yes | Yes | Yes | Yes | Yes | Yes | Yes | Yes | Yes |
| PUNTUACIÓN JBI | 9/10 | 9/10 | 10/10 | 9/10 | 7/10 | 9/10 | 10/10 | 8/10 | 10/10 | 9/10 | 8/10 |

**Table S2.** CERQual assessments.

| **Summary of the review search** | **Reports of studies contributing to the review finding** | **CERQual assessment of confidence in the evidence** | **Explanation of CERQual assessment** |
| --- | --- | --- | --- |
| Adaptation to Digital Health in Primary Care.  - Need for rapid adaptation and change.  - Patient safety.  - Logistical challenges. | Carlsson et al., 2022; Entezarjou et al., 2020; Havard et al., 2024; Nilsen et al., 2022; Navarro-Martínez et al., 2024. | High confidence | 4 studies with moderate methodological limitations, 1 study with moderate relevance concerns. No concerns about data consistency and adequacy. |
| Digital Health from the perspective of Primary Care nurses.  - Technology management and new working methods.  - Need for training in Digital Health.  - Benefits of Digital Health.  - Challenges of Digital Health. | Ashley et al., 2023; Carlsson et al., 2022; Ekstedt et al., 2023; James et al., 2021; Navarro-Martínez et al., 2024. | High confidence | 4 studies with moderate methodological limitations. No concerns about relevance, consistency and adequacy of data. |
| Digital nurse-patient interaction.  - Evolution of care through virtual interactions.  - Advantages of virtual care.  - Difficulties of virtual care. | Ashley et al., 2023; Carlsson et al., 2022; Ekstedt et al., 2023; James et al., 2021; Lie et al., 2019; Navarro-Martínez et al., 2024; Nilsen et al., 2022; Rouleau et al. 2024; Van Grootven et al., 2023. | Moderate confidence | 5 studies with moderate methodological limitations, 2 studies with moderate consistency concerns No concerns about relevance and adequacy of data. |
